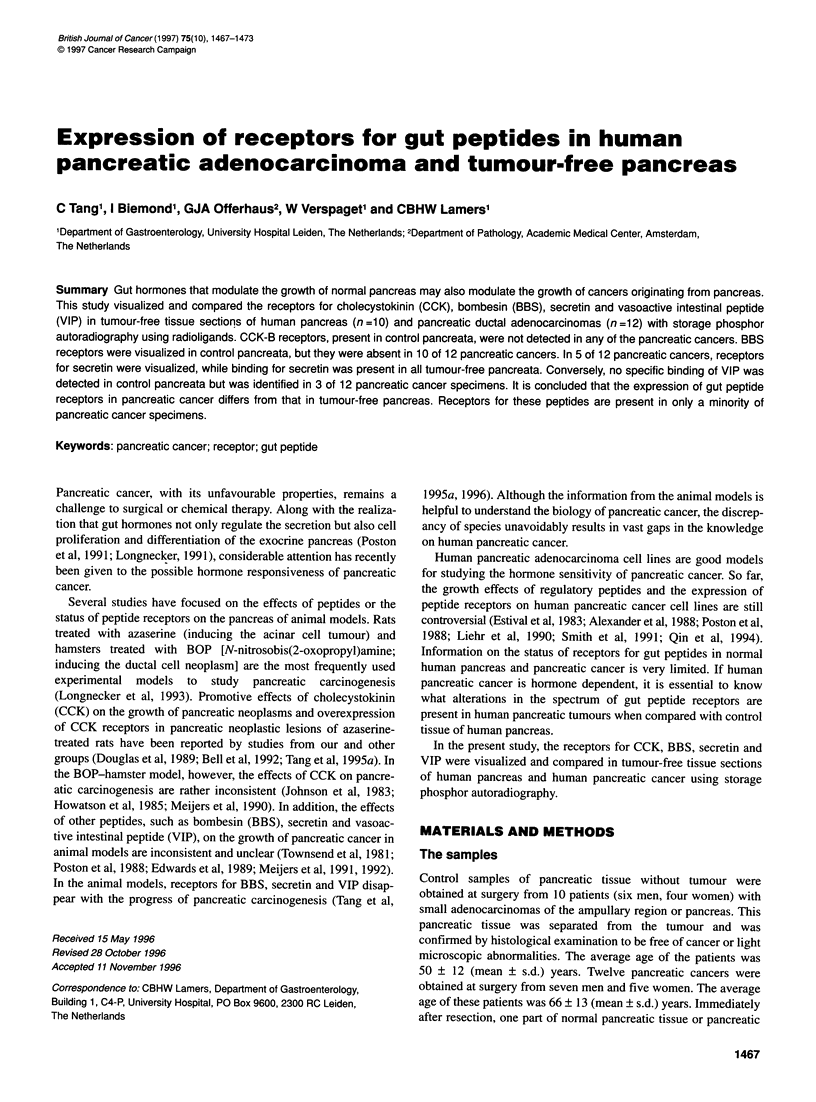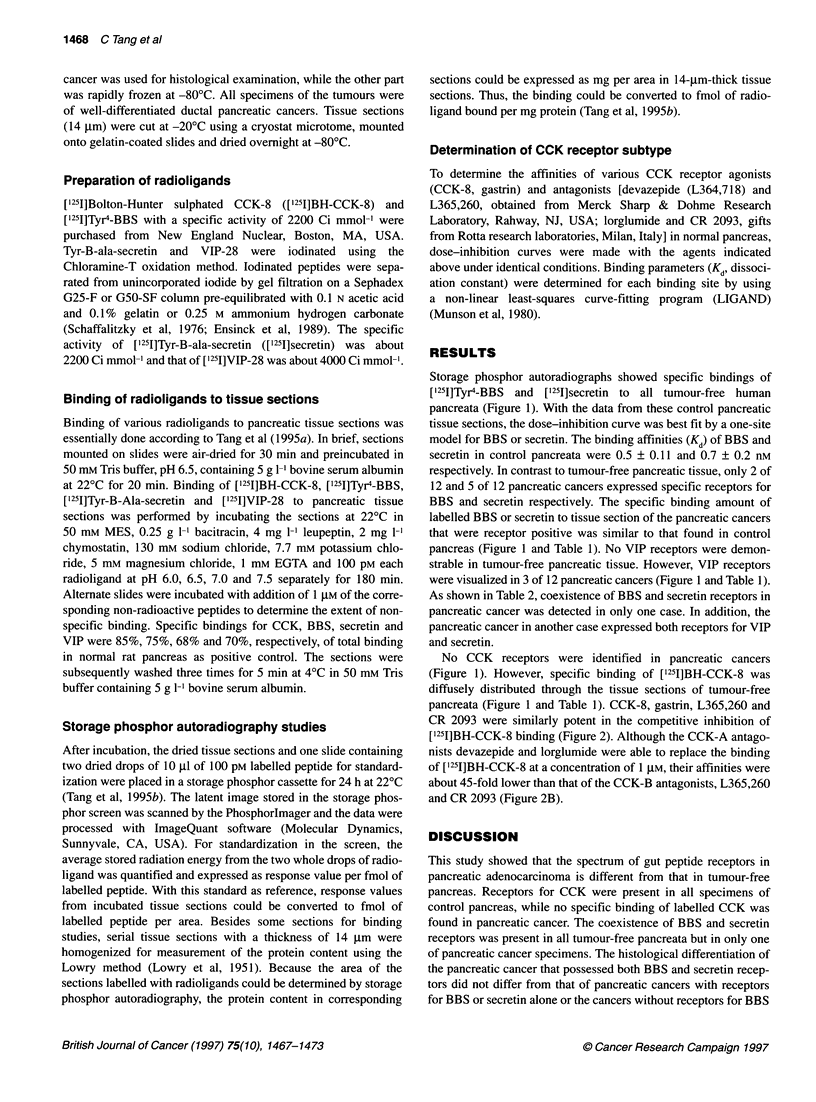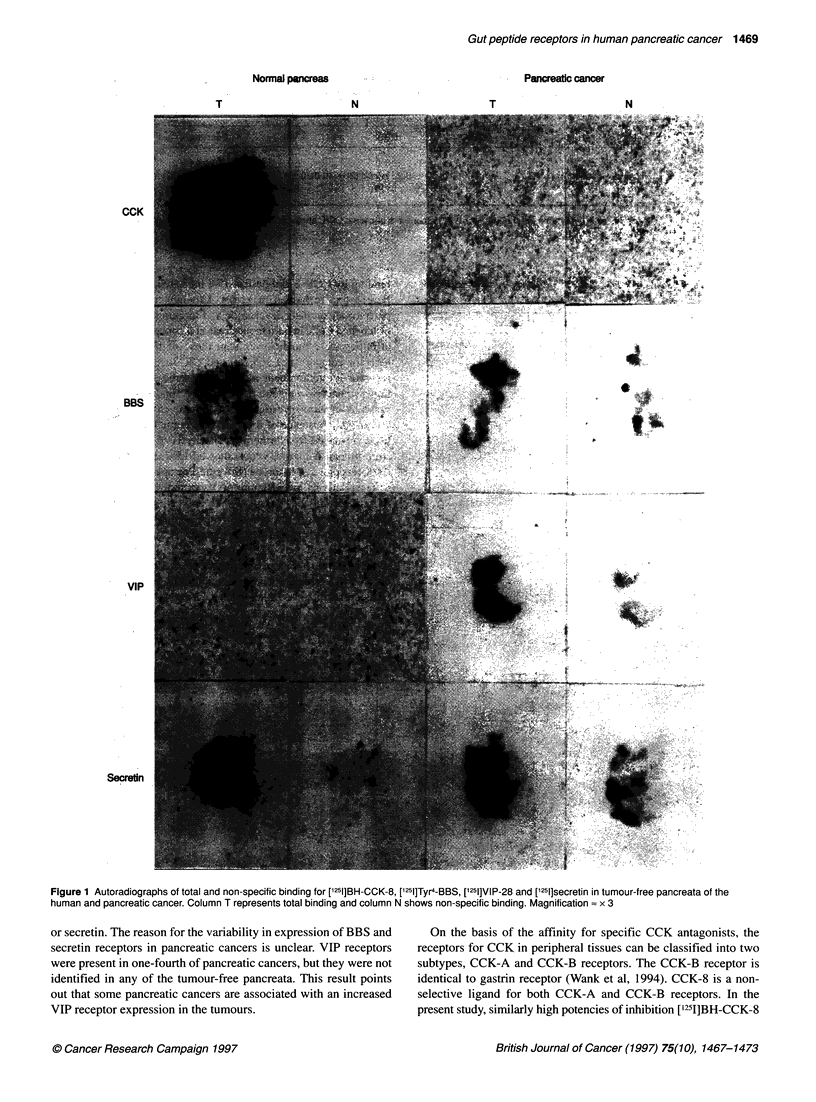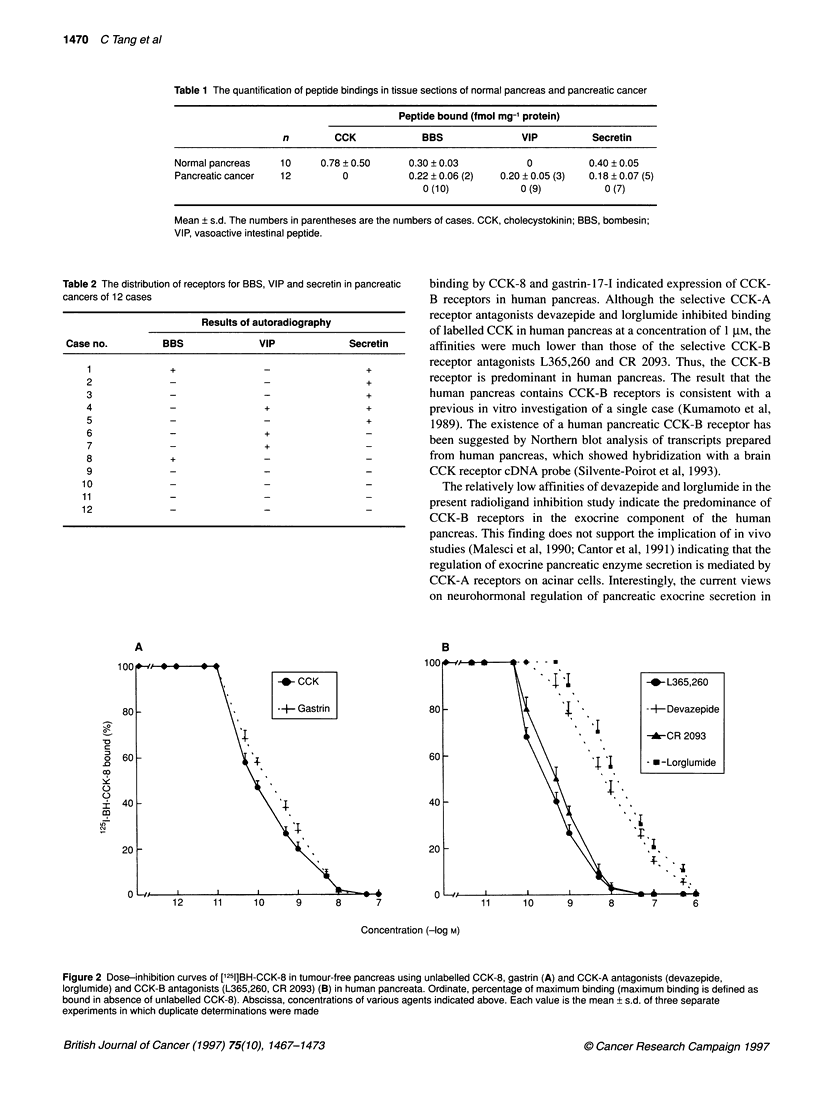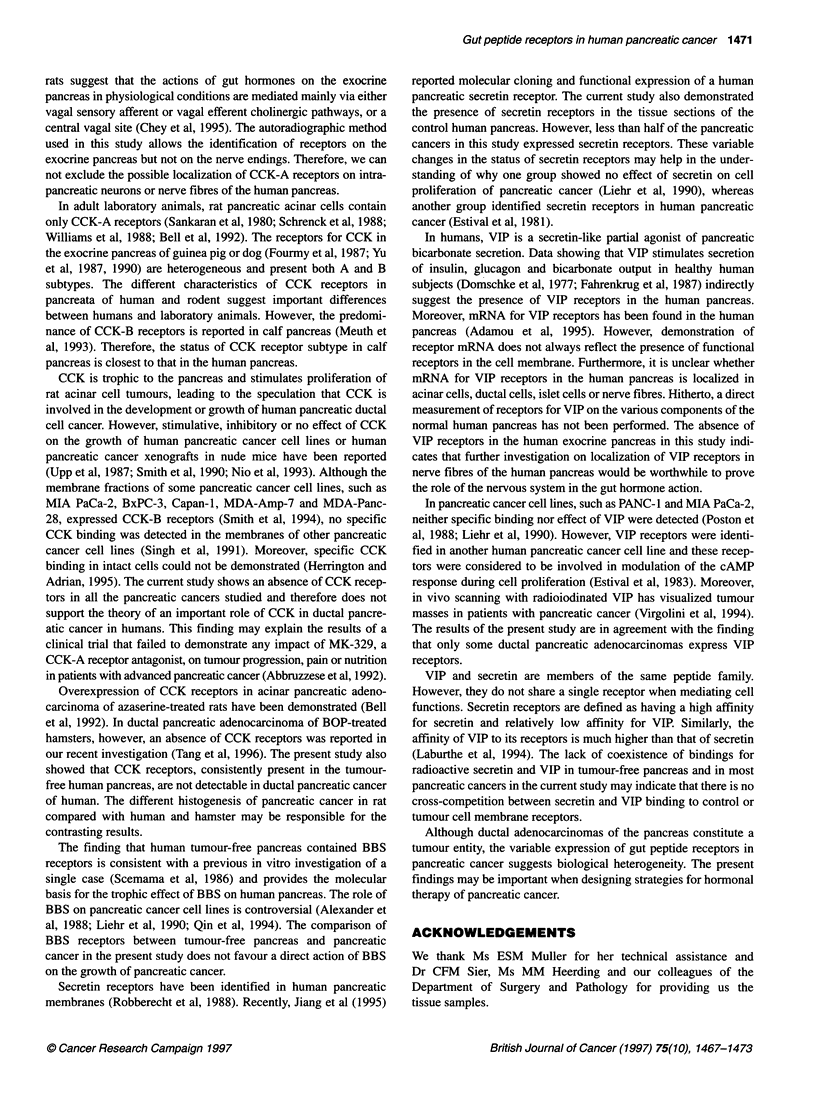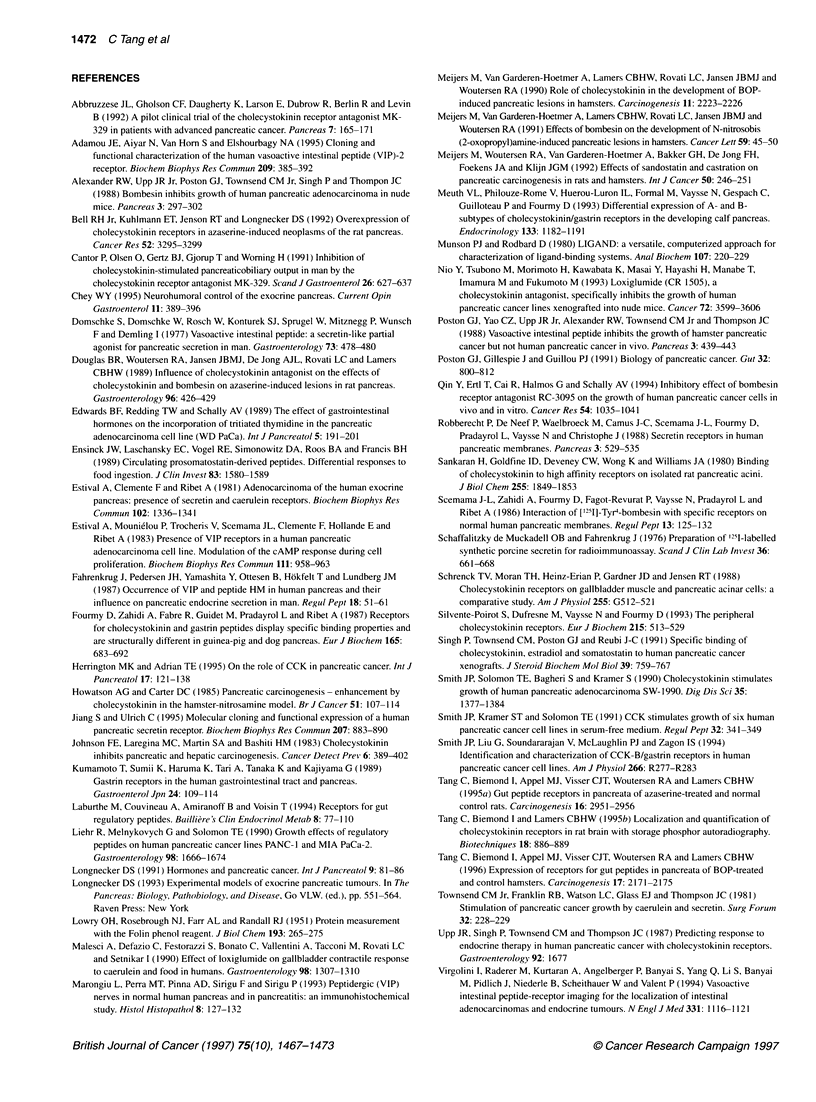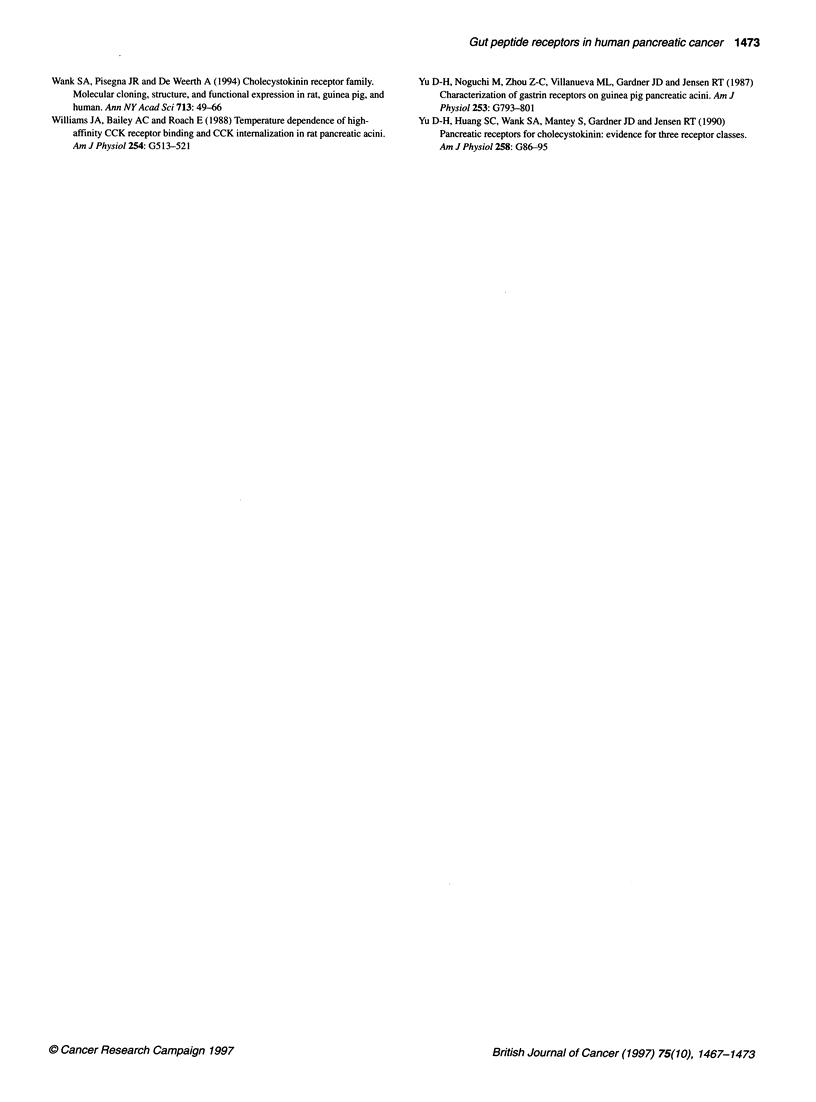# Expression of receptors for gut peptides in human pancreatic adenocarcinoma and tumour-free pancreas.

**DOI:** 10.1038/bjc.1997.251

**Published:** 1997

**Authors:** C. Tang, I. Biemond, G. J. Offerhaus, W. Verspaget, C. B. Lamers

**Affiliations:** Department of Gastroenterology, University Hospital Leiden, The Netherlands.

## Abstract

**Images:**


British Journal of Cancer (1997) 75(10), 1467–1473
© 1997 Cancer Research Campaign

### Expression of receptors for gut peptides in human pancreatic adenocarcinoma and tumour-free pancreas

C Tang[1], I Biemond[1], GJA Offerhaus[2], W Verspaget[1] and CBHW Lamers[1]

[1]Department of Gastroenterology, University Hospital Leiden, The Netherlands; [2]Department of Pathology, Academic Medical Center, Amsterdam, The Netherlands

**Summary** Gut hormones that modulate the growth of normal pancreas may also modulate the growth of cancers originating from pancreas. This study visualized and compared the receptors for cholecystokinin (CCK), bombesin (BBS), secretin and vasoactive intestinal peptide (VIP) in tumour-free tissue sections of human pancreas ($n$ =10) and pancreatic ductal adenocarcinomas ($n$ =12) with storage phosphor autoradiography using radioligands. CCK-B receptors, present in control pancreata, were not detected in any of the pancreatic cancers. BBS receptors were visualized in control pancreata, but they were absent in 10 of 12 pancreatic cancers. In 5 of 12 pancreatic cancers, receptors for secretin were visualized, while binding for secretin was present in all tumour-free pancreata. Conversely, no specific binding of VIP was detected in control pancreata but was identified in 3 of 12 pancreatic cancer specimens. It is concluded that the expression of gut peptide receptors in pancreatic cancer differs from that in tumour-free pancreas. Receptors for these peptides are present in only a minority of pancreatic cancer specimens.

**Keywords**: pancreatic cancer; receptor; gut peptide

Pancreatic cancer, with its unfavourable properties, remains a challenge to surgical or chemical therapy. Along with the realization that gut hormones not only regulate the secretion but also cell proliferation and differentiation of the exocrine pancreas (Poston et al, 1991; Longnecker, 1991), considerable attention has recently been given to the possible hormone responsiveness of pancreatic cancer.

Several studies have focused on the effects of peptides or the status of peptide receptors on the pancreas of animal models. Rats treated with azaserine (inducing the acinar cell tumour) and hamsters treated with BOP [*N*-nitrosobis(2-oxopropyl)amine; inducing the ductal cell neoplasm] are the most frequently used experimental models to study pancreatic carcinogenesis (Longnecker et al, 1993). Promotive effects of cholecystokinin (CCK) on the growth of pancreatic neoplasms and overexpression of CCK receptors in pancreatic neoplastic lesions of azaserine-treated rats have been reported by studies from our and other groups (Douglas et al, 1989; Bell et al, 1992; Tang et al, 1995*a*). In the BOP–hamster model, however, the effects of CCK on pancreatic carcinogenesis are rather inconsistent (Johnson et al, 1983; Howatson et al, 1985; Meijers et al, 1990). In addition, the effects of other peptides, such as bombesin (BBS), secretin and vasoactive intestinal peptide (VIP), on the growth of pancreatic cancer in animal models are inconsistent and unclear (Townsend et al, 1981; Poston et al, 1988; Edwards et al, 1989; Meijers et al, 1991, 1992). In the animal models, receptors for BBS, secretin and VIP disappear with the progress of pancreatic carcinogenesis (Tang et al, 1995*a*, 1996). Although the information from the animal models is helpful to understand the biology of pancreatic cancer, the discrepancy of species unavoidably results in vast gaps in the knowledge on human pancreatic cancer.

Human pancreatic adenocarcinoma cell lines are good models for studying the hormone sensitivity of pancreatic cancer. So far, the growth effects of regulatory peptides and the expression of peptide receptors on human pancreatic cancer cell lines are still controversial (Estival et al, 1983; Alexander et al, 1988; Poston et al, 1988; Liehr et al, 1990; Smith et al, 1991; Qin et al, 1994). Information on the status of receptors for gut peptides in normal human pancreas and pancreatic cancer is very limited. If human pancreatic cancer is hormone dependent, it is essential to know what alterations in the spectrum of gut peptide receptors are present in human pancreatic tumours when compared with control tissue of human pancreas.

In the present study, the receptors for CCK, BBS, secretin and VIP were visualized and compared in tumour-free tissue sections of human pancreas and human pancreatic cancer using storage phosphor autoradiography.

### MATERIALS AND METHODS

### The samples

Control samples of pancreatic tissue without tumour were obtained at surgery from 10 patients (six men, four women) with small adenocarcinomas of the ampullary region or pancreas. This pancreatic tissue was separated from the tumour and was confirmed by histological examination to be free of cancer or light microscopic abnormalities. The average age of the patients was 50 ± 12 (mean ± s.d.) years. Twelve pancreatic cancers were obtained at surgery from seven men and five women. The average age of these patients was 66 ± 13 (mean ± s.d.) years. Immediately after resection, one part of normal pancreatic tissue or pancreatic

*Received 15 May 1996*
*Revised 28 October 1996*
*Accepted 11 November 1996*

*Correspondence to:* CBHW Lamers, Department of Gastroenterology, Building 1, C4-P, University Hospital, PO Box 9600, 2300 RC Leiden, The Netherlands cancer was used for histological examination, while the other part was rapidly frozen at –80°C. All specimens of the tumours were of well-differentiated ductal pancreatic cancers. Tissue sections (14 μm) were cut at –20°C using a cryostat microtome, mounted onto gelatin-coated slides and dried overnight at –80°C.

### Preparation of radioligands

[$^{125}$I]Bolton-Hunter sulphated CCK-8 ([$^{125}$I]BH-CCK-8) and [$^{125}$I]Tyr$^4$-BBS with a specific activity of 2200 Ci mmol$^{-1}$ were purchased from New England Nuclear, Boston, MA, USA. Tyr-B-ala-secretin and VIP-28 were iodinated using the Chloramine-T oxidation method. Iodinated peptides were separated from unincorporated iodide by gel filtration on a Sephadex G25-F or G50-SF column pre-equilibrated with 0.1 N acetic acid and 0.1% gelatin or 0.25 M ammonium hydrogen carbonate (Schaffalitzky et al, 1976; Ensinck et al, 1989). The specific activity of [$^{125}$I]Tyr-B-ala-secretin ([$^{125}$I]secretin) was about 2200 Ci mmol$^{-1}$ and that of [$^{125}$I]VIP-28 was about 4000 Ci mmol$^{-1}$.

### Binding of radioligands to tissue sections

Binding of various radioligands to pancreatic tissue sections was essentially done according to Tang et al (1995*a*). In brief, sections mounted on slides were air-dried for 30 min and preincubated in 50 mM Tris buffer, pH 6.5, containing 5 g l$^{-1}$ bovine serum albumin at 22°C for 20 min. Binding of [$^{125}$I]BH-CCK-8, [$^{125}$I]Tyr$^4$-BBS, [$^{125}$I]Tyr-B-Ala-secretin and [$^{125}$I]VIP-28 to pancreatic tissue sections was performed by incubating the sections at 22°C in 50 mM MES, 0.25 g l$^{-1}$ bacitracin, 4 mg l$^{-1}$ leupeptin, 2 mg l$^{-1}$ chymostatin, 130 mM sodium chloride, 7.7 mM potassium chloride, 5 mM magnesium chloride, 1 mM EGTA and 100 pM each radioligand at pH 6.0, 6.5, 7.0 and 7.5 separately for 180 min. Alternate slides were incubated with addition of 1 μM of the corresponding non-radioactive peptides to determine the extent of non-specific binding. Specific bindings for CCK, BBS, secretin and VIP were 85%, 75%, 68% and 70%, respectively, of total binding in normal rat pancreas as positive control. The sections were subsequently washed three times for 5 min at 4°C in 50 mM Tris buffer containing 5 g l$^{-1}$ bovine serum albumin.

### Storage phosphor autoradiography studies

After incubation, the dried tissue sections and one slide containing two dried drops of 10 μl of 100 pM labelled peptide for standardization were placed in a storage phosphor cassette for 24 h at 22°C (Tang et al, 1995*b*). The latent image stored in the storage phosphor screen was scanned by the PhosphorImager and the data were processed with ImageQuant software (Molecular Dynamics, Sunnyvale, CA, USA). For standardization in the screen, the average stored radiation energy from the two whole drops of radioligand was quantified and expressed as response value per fmol of labelled peptide. With this standard as reference, response values from incubated tissue sections could be converted to fmol of labelled peptide per area. Besides some sections for binding studies, serial tissue sections with a thickness of 14 μm were homogenized for measurement of the protein content using the Lowry method (Lowry et al, 1951). Because the area of the sections labelled with radioligands could be determined by storage phosphor autoradiography, the protein content in corresponding sections could be expressed as mg per area in 14-μm-thick tissue sections. Thus, the binding could be converted to fmol of radioligand bound per mg protein (Tang et al, 1995*b*).

### Determination of CCK receptor subtype

To determine the affinities of various CCK receptor agonists (CCK-8, gastrin) and antagonists [devazepide (L364,718) and L365,260, obtained from Merck Sharp & Dohme Research Laboratory, Rahway, NJ, USA; lorglumide and CR 2093, gifts from Rotta research laboratories, Milan, Italy] in normal pancreas, dose–inhibition curves were made with the agents indicated above under identical conditions. Binding parameters ($K_d$, dissociation constant) were determined for each binding site by using a non-linear least-squares curve-fitting program (LIGAND) (Munson et al, 1980).

### RESULTS

Storage phosphor autoradiographs showed specific bindings of [$^{125}$I]Tyr$^4$-BBS and [$^{125}$I]secretin to all tumour-free human pancreata (Figure 1). With the data from these control pancreatic tissue sections, the dose–inhibition curve was best fit by a one-site model for BBS or secretin. The binding affinities ($K_d$) of BBS and secretin in control pancreata were 0.5 ± 0.11 and 0.7 ± 0.2 nM respectively. In contrast to tumour-free pancreatic tissue, only 2 of 12 and 5 of 12 pancreatic cancers expressed specific receptors for BBS and secretin respectively. The specific binding amount of labelled BBS or secretin to tissue section of the pancreatic cancers that were receptor positive was similar to that found in control pancreas (Figure 1 and Table 1). No VIP receptors were demonstrable in tumour-free pancreatic tissue. However, VIP receptors were visualized in 3 of 12 pancreatic cancers (Figure 1 and Table 1). As shown in Table 2, coexistence of BBS and secretin receptors in pancreatic cancer was detected in only one case. In addition, the pancreatic cancer in another case expressed both receptors for VIP and secretin.

No CCK receptors were identified in pancreatic cancers (Figure 1). However, specific binding of [$^{125}$I]BH-CCK-8 was diffusely distributed through the tissue sections of tumour-free pancreata (Figure 1 and Table 1). CCK-8, gastrin, L365,260 and CR 2093 were similarly potent in the competitive inhibition of [$^{125}$I]BH-CCK-8 binding (Figure 2). Although the CCK-A antagonists devazepide and lorglumide were able to replace the binding of [$^{125}$I]BH-CCK-8 at a concentration of 1 μM, their affinities were about 45-fold lower than that of the CCK-B antagonists, L365,260 and CR 2093 (Figure 2B).

### DISCUSSION

This study showed that the spectrum of gut peptide receptors in pancreatic adenocarcinoma is different from that in tumour-free pancreas. Receptors for CCK were present in all specimens of control pancreas, while no specific binding of labelled CCK was found in pancreatic cancer. The coexistence of BBS and secretin receptors was present in all tumour-free pancreata but in only one of pancreatic cancer specimens. The histological differentiation of the pancreatic cancer that possessed both BBS and secretin receptors did not differ from that of pancreatic cancers with receptors for BBS or secretin alone or the cancers without receptors for BBS

 © Cancer Research Campaign 1997

**Figure 1** Autoradiographs of total and non-specific binding for [$^{125}$I]BH-CCK-8, [$^{125}$I]Tyr$^4$-BBS, [$^{125}$I]VIP-28 and [$^{125}$I]secretin in tumour-free pancreata of the human and pancreatic cancer. Column T represents total binding and column N shows non-specific binding. Magnification ≈ × 3

or secretin. The reason for the variability in expression of BBS and secretin receptors in pancreatic cancers is unclear. VIP receptors were present in one-fourth of pancreatic cancers, but they were not identified in any of the tumour-free pancreata. This result points out that some pancreatic cancers are associated with an increased VIP receptor expression in the tumours.

On the basis of the affinity for specific CCK antagonists, the receptors for CCK in peripheral tissues can be classified into two subtypes, CCK-A and CCK-B receptors. The CCK-B receptor is identical to gastrin receptor (Wank et al, 1994). CCK-8 is a non-selective ligand for both CCK-A and CCK-B receptors. In the present study, similarly high potencies of inhibition [$^{125}$I]BH-CCK-8

*© Cancer Research Campaign 1997*

**Table 1** The quantification of peptide bindings in tissue sections of normal pancreas and pancreatic cancer

| | | Peptide bound (fmol mg$^{-1}$ protein) | | | |
|---|---|---|---|---|---|
| | *n* | CCK | BBS | VIP | Secretin |
| Normal pancreas | 10 | 0.78 ± 0.50 | 0.30 ± 0.03 | 0 | 0.40 ± 0.05 |
| Pancreatic cancer | 12 | 0 | 0.22 ± 0.06 (2) | 0.20 ± 0.05 (3) | 0.18 ± 0.07 (5) |
| | | | 0 (10) | 0 (9) | 0 (7) |

Mean ± s.d. The numbers in parentheses are the numbers of cases. CCK, cholecystokinin; BBS, bombesin; VIP, vasoactive intestinal peptide.

**Table 2** The distribution of receptors for BBS, VIP and secretin in pancreatic cancers of 12 cases

| | Results of autoradiography | | |
|---|---|---|---|
| Case no. | BBS | VIP | Secretin |
| 1 | + | – | + |
| 2 | – | – | + |
| 3 | – | – | + |
| 4 | – | + | + |
| 5 | – | – | + |
| 6 | – | + | – |
| 7 | – | + | – |
| 8 | + | – | – |
| 9 | – | – | – |
| 10 | – | – | – |
| 11 | – | – | – |
| 12 | – | – | – |

binding by CCK-8 and gastrin-17-I indicated expression of CCK-B receptors in human pancreas. Although the selective CCK-A receptor antagonists devazepide and lorglumide inhibited binding of labelled CCK in human pancreas at a concentration of 1 μM, the affinities were much lower than those of the selective CCK-B receptor antagonists L365,260 and CR 2093. Thus, the CCK-B receptor is predominant in human pancreas. The result that the human pancreas contains CCK-B receptors is consistent with a previous in vitro investigation of a single case (Kumamoto et al, 1989). The existence of a human pancreatic CCK-B receptor has been suggested by Northern blot analysis of transcripts prepared from human pancreas, which showed hybridization with a brain CCK receptor cDNA probe (Silvente-Poirot et al, 1993).

The relatively low affinities of devazepide and lorglumide in the present radioligand inhibition study indicate the predominance of CCK-B receptors in the exocrine component of the human pancreas. This finding does not support the implication of in vivo studies (Malesci et al, 1990; Cantor et al, 1991) indicating that the regulation of exocrine pancreatic enzyme secretion is mediated by CCK-A receptors on acinar cells. Interestingly, the current views on neurohormonal regulation of pancreatic exocrine secretion in

**Figure 2** Dose–inhibition curves of [$^{125}$I]BH-CCK-8 in tumour-free pancreas using unlabelled CCK-8, gastrin (**A**) and CCK-A antagonists (devazepide, lorglumide) and CCK-B antagonists (L365,260, CR 2093) (**B**) in human pancreata. Ordinate, percentage of maximum binding (maximum binding is defined as bound in absence of unlabelled CCK-8). Abscissa, concentrations of various agents indicated above. Each value is the mean ± s.d. of three separate experiments in which duplicate determinations were made

 © Cancer Research Campaign 1997

rats suggest that the actions of gut hormones on the exocrine pancreas in physiological conditions are mediated mainly via either vagal sensory afferent or vagal efferent cholinergic pathways, or a central vagal site (Chey et al, 1995). The autoradiographic method used in this study allows the identification of receptors on the exocrine pancreas but not on the nerve endings. Therefore, we can not exclude the possible localization of CCK-A receptors on intrapancreatic neurons or nerve fibres of the human pancreas.

In adult laboratory animals, rat pancreatic acinar cells contain only CCK-A receptors (Sankaran et al, 1980; Schrenck et al, 1988; Williams et al, 1988; Bell et al, 1992). The receptors for CCK in the exocrine pancreas of guinea pig or dog (Fourmy et al, 1987; Yu et al, 1987, 1990) are heterogeneous and present both A and B subtypes. The different characteristics of CCK receptors in pancreata of human and rodent suggest important differences between humans and laboratory animals. However, the predominance of CCK-B receptors is reported in calf pancreas (Meuth et al, 1993). Therefore, the status of CCK receptor subtype in calf pancreas is closest to that in the human pancreas.

CCK is trophic to the pancreas and stimulates proliferation of rat acinar cell tumours, leading to the speculation that CCK is involved in the development or growth of human pancreatic ductal cell cancer. However, stimulative, inhibitory or no effect of CCK on the growth of human pancreatic cancer cell lines or human pancreatic cancer xenografts in nude mice have been reported (Upp et al, 1987; Smith et al, 1990; Nio et al, 1993). Although the membrane fractions of some pancreatic cancer cell lines, such as MIA PaCa-2, BxPC-3, Capan-1, MDA-Amp-7 and MDA-Panc-28, expressed CCK-B receptors (Smith et al, 1994), no specific CCK binding was detected in the membranes of other pancreatic cancer cell lines (Singh et al, 1991). Moreover, specific CCK binding in intact cells could not be demonstrated (Herrington and Adrian, 1995). The current study shows an absence of CCK receptors in all the pancreatic cancers studied and therefore does not support the theory of an important role of CCK in ductal pancreatic cancer in humans. This finding may explain the results of a clinical trial that failed to demonstrate any impact of MK-329, a CCK-A receptor antagonist, on tumour progression, pain or nutrition in patients with advanced pancreatic cancer (Abbruzzese et al, 1992).

Overexpression of CCK receptors in acinar pancreatic adenocarcinoma of azaserine-treated rats have been demonstrated (Bell et al, 1992). In ductal pancreatic adenocarcinoma of BOP-treated hamsters, however, an absence of CCK receptors was reported in our recent investigation (Tang et al, 1996). The present study also showed that CCK receptors, consistently present in the tumour-free human pancreas, are not detectable in ductal pancreatic cancer of human. The different histogenesis of pancreatic cancer in rat compared with human and hamster may be responsible for the contrasting results.

The finding that human tumour-free pancreas contained BBS receptors is consistent with a previous in vitro investigation of a single case (Scemama et al, 1986) and provides the molecular basis for the trophic effect of BBS on human pancreas. The role of BBS on pancreatic cancer cell lines is controversial (Alexander et al, 1988; Liehr et al, 1990; Qin et al, 1994). The comparison of BBS receptors between tumour-free pancreas and pancreatic cancer in the present study does not favour a direct action of BBS on the growth of pancreatic cancer.

Secretin receptors have been identified in human pancreatic membranes (Robberecht et al, 1988). Recently, Jiang et al (1995) reported molecular cloning and functional expression of a human pancreatic secretin receptor. The current study also demonstrated the presence of secretin receptors in the tissue sections of the control human pancreas. However, less than half of the pancreatic cancers in this study expressed secretin receptors. These variable changes in the status of secretin receptors may help in the understanding of why one group showed no effect of secretin on cell proliferation of pancreatic cancer (Liehr et al, 1990), whereas another group identified secretin receptors in human pancreatic cancer (Estival et al, 1981).

In humans, VIP is a secretin-like partial agonist of pancreatic bicarbonate secretion. Data showing that VIP stimulates secretion of insulin, glucagon and bicarbonate output in healthy human subjects (Domschke et al, 1977; Fahrenkrug et al, 1987) indirectly suggest the presence of VIP receptors in the human pancreas. Moreover, mRNA for VIP receptors has been found in the human pancreas (Adamou et al, 1995). However, demonstration of receptor mRNA does not always reflect the presence of functional receptors in the cell membrane. Furthermore, it is unclear whether mRNA for VIP receptors in the human pancreas is localized in acinar cells, ductal cells, islet cells or nerve fibres. Hitherto, a direct measurement of receptors for VIP on the various components of the normal human pancreas has not been performed. The absence of VIP receptors in the human exocrine pancreas in this study indicates that further investigation on localization of VIP receptors in nerve fibres of the human pancreas would be worthwhile to prove the role of the nervous system in the gut hormone action.

In pancreatic cancer cell lines, such as PANC-1 and MIA PaCa-2, neither specific binding nor effect of VIP were detected (Poston et al, 1988; Liehr et al, 1990). However, VIP receptors were identified in another human pancreatic cancer cell line and these receptors were considered to be involved in modulation of the cAMP response during cell proliferation (Estival et al, 1983). Moreover, in vivo scanning with radioiodinated VIP has visualized tumour masses in patients with pancreatic cancer (Virgolini et al, 1994). The results of the present study are in agreement with the finding that only some ductal pancreatic adenocarcinomas express VIP receptors.

VIP and secretin are members of the same peptide family. However, they do not share a single receptor when mediating cell functions. Secretin receptors are defined as having a high affinity for secretin and relatively low affinity for VIP. Similarly, the affinity of VIP to its receptors is much higher than that of secretin (Laburthe et al, 1994). The lack of coexistence of bindings for radioactive secretin and VIP in tumour-free pancreas and in most pancreatic cancers in the current study may indicate that there is no cross-competition between secretin and VIP binding to control or tumour cell membrane receptors.

Although ductal adenocarcinomas of the pancreas constitute a tumour entity, the variable expression of gut peptide receptors in pancreatic cancer suggests biological heterogeneity. The present findings may be important when designing strategies for hormonal therapy of pancreatic cancer.

### ACKNOWLEDGEMENTS

We thank Ms ESM Muller for her technical assistance and Dr CFM Sier, Ms MM Heerding and our colleagues of the Department of Surgery and Pathology for providing us the tissue samples.

### REFERENCES

Abbruzzese JL, Gholson CF, Daugherty K, Larson E, Dubrow R, Berlin R and Levin B (1992) A pilot clinical trial of the cholecystokinin receptor antagonist MK-329 in patients with advanced pancreatic cancer. *Pancreas* **7**: 165–171

Adamou JE, Aiyar N, Van Horn S and Elshourbagy NA (1995) Cloning and functional characterization of the human vasoactive intestinal peptide (VIP)-2 receptor. *Biochem Biophys Res Commun* **209**: 385–392

Alexander RW, Upp JR Jr, Poston GJ, Townsend CM Jr, Singh P and Thompon JC (1988) Bombesin inhibits growth of human pancreatic adenocarcinoma in nude mice. *Pancreas* **3**: 297–302

Bell RH Jr, Kuhlmann ET, Jenson RT and Longnecker DS (1992) Overexpression of cholecystokinin receptors in azaserine-induced neoplasms of the rat pancreas. *Cancer Res* **52**: 3295–3299

Cantor P, Olsen O, Gertz BJ, Gjorup T and Worning H (1991) Inhibition of cholecystokinin-stimulated pancreaticobiliary output in man by the cholecystokinin receptor antagonist MK-329. *Scand J Gastroenterol* **26**: 627–637

Chey WY (1995) Neurohumoral control of the exocrine pancreas. *Current Opin Gastroenterol* **11**: 389–396

Domschke S, Domschke W, Rosch W, Konturek SJ, Sprugel W, Mitznegg P, Wunsch F and Demling I (1977) Vasoactive intestinal peptide: a secretin-like partial agonist for pancreatic secretion in man. *Gastroenterology* **73**: 478–480

Douglas BR, Woutersen RA, Jansen JBMJ, De Jong AJL, Rovati LC and Lamers CBHW (1989) Influence of cholecystokinin antagonist on the effects of cholecystokinin and bombesin on azaserine-induced lesions in rat pancreas. *Gastroenterology* **96**: 426–429

Edwards BF, Redding TW and Schally AV (1989) The effect of gastrointestinal hormones on the incorporation of tritiated thymidine in the pancreatic adenocarcinoma cell line (WD PaCa). *Int J Pancreatol* **5**: 191–201

Ensinck JW, Laschansky EC, Vogel RE, Simonowitz DA, Roos BA and Francis BH (1989) Circulating prosomatostatin-derived peptides. Differential responses to food ingestion. *J Clin Invest* **83**: 1580–1589

Estival A, Clemente F and Ribet A (1981) Adenocarcinoma of the human exocrine pancreas: presence of secretin and caerulein receptors. *Biochem Biophys Res Commun* **102**: 1336–1341

Estival A, Mouniélou P, Trocheris V, Scemama JL, Clemente F, Hollande E and Ribet A (1983) Presence of VIP receptors in a human pancreatic adenocarcinoma cell line. Modulation of the cAMP response during cell proliferation. *Biochem Biophys Res Commun* **111**: 958–963

Fahrenkrug J, Pedersen JH, Yamashita Y, Ottesen B, Hökfelt T and Lundberg JM (1987) Occurrence of VIP and peptide HM in human pancreas and their influence on pancreatic endocrine secretion in man. *Regul Pept* **18**: 51–61

Fourmy D, Zahidi A, Fabre R, Guidet M, Pradayrol L and Ribet A (1987) Receptors for cholecystokinin and gastrin peptides display specific binding properties and are structurally different in guinea-pig and dog pancreas. *Eur J Biochem* **165**: 683–692

Herrington MK and Adrian TE (1995) On the role of CCK in pancreatic cancer. *Int J Pancreatol* **17**: 121–138

Howatson AG and Carter DC (1985) Pancreatic carcinogenesis – enhancement by cholecystokinin in the hamster-nitrosamine model. *Br J Cancer* **51**: 107–114

Jiang S and Ulrich C (1995) Molecular cloning and functional expression of a human pancreatic secretin receptor. *Biochem Biophys Res Commun* **207**: 883–890

Johnson FE, Laregina MC, Martin SA and Bashiti HM (1983) Cholecystokinin inhibits pancreatic and hepatic carcinogenesis. *Cancer Detect Prev* **6**: 389–402

Kumamoto T, Sumii K, Haruma K, Tari A, Tanaka K and Kajiyama G (1989) Gastrin receptors in the human gastrointestinal tract and pancreas. *Gastroenterol Jpn* **24**: 109–114

Laburthe M, Couvineau A, Amiranoff B and Voisin T (1994) Receptors for gut regulatory peptides. *Baillière's Clin Endocrinol Metab* **8**: 77–110

Liehr R, Melnykovych G and Solomon TE (1990) Growth effects of regulatory peptides on human pancreatic cancer lines PANC-1 and MIA PaCa-2. *Gastroenterology* **98**: 1666–1674

Longnecker DS (1991) Hormones and pancreatic cancer. *Int J Pancreatol* **9**: 81–86

Longnecker DS (1993) Experimental models of exocrine pancreatic tumours. In *The Pancreas: Biology, Pathobiology, and Disease*, Go VLW. (ed.), pp. 551–564. Raven Press: New York

Lowry OH, Rosebrough NJ, Farr AL and Randall RJ (1951) Protein measurement with the Folin phenol reagent. *J Biol Chem* **193**: 265–275

Malesci A, Defazio C, Festorazzi S, Bonato C, Vallentini A, Tacconi M, Rovati LC and Setnikar I (1990) Effect of loxiglumide on gallbladder contractile response to caerulein and food in humans. *Gastroenterology* **98**: 1307–1310

Marongiu L, Perra MT, Pinna AD, Sirigu F and Sirigu P (1993) Peptidergic (VIP) nerves in normal human pancreas and in pancreatitis: an immunohistochemical study. *Histol Histopathol* **8**: 127–132

Meijers M, Van Garderen-Hoetmer A, Lamers CBHW, Rovati LC, Jansen JBMJ and Woutersen RA (1990) Role of cholecystokinin in the development of BOP-induced pancreatic lesions in hamsters. *Carcinogenesis* **11**: 2223–2226

Meijers M, Van Garderen-Hoetmer A, Lamers CBHW, Rovati LC, Jansen JBMJ and Woutersen RA (1991) Effects of bombesin on the development of N-nitrosobis (2-oxopropyl)amine-induced pancreatic lesions in hamsters. *Cancer Lett* **59**: 45–50

Meijers M, Woutersen RA, Van Garderen-Hoetmer A, Bakker GH, De Jong FH, Foekens JA and Klijn JGM (1992) Effects of sandostatin and castration on pancreatic carcinogenesis in rats and hamsters. *Int J Cancer* **50**: 246–251

Meuth VL, Philouze-Rome V, Huerou-Luron IL, Formal M, Vaysse N, Gespach C, Guilloteau P and Fourmy D (1993) Differential expression of A- and B-subtypes of cholecystokinin/gastrin receptors in the developing calf pancreas. *Endocrinology* **133**: 1182–1191

Munson PJ and Rodbard D (1980) LIGAND: a versatile, computerized approach for characterization of ligand-binding systems. *Anal Biochem* **107**: 220–229

Nio Y, Tsubono M, Morimoto H, Kawabata K, Masai Y, Hayashi H, Manabe T, Imamura M and Fukumoto M (1993) Loxiglumide (CR 1505), a cholecystokinin antagonist, specifically inhibits the growth of human pancreatic cancer lines xenografted into nude mice. *Cancer* **72**: 3599–3606

Poston GJ, Yao CZ, Upp JR Jr, Alexander RW, Townsend CM Jr and Thompson JC (1988) Vasoactive intestinal peptide inhibits the growth of hamster pancreatic cancer but not human pancreatic cancer in vivo. *Pancreas* **3**: 439–443

Poston GJ, Gillespie J and Guillou PJ (1991) Biology of pancreatic cancer. *Gut* **32**: 800–812

Qin Y, Ertl T, Cai R, Halmos G and Schally AV (1994) Inhibitory effect of bombesin receptor antagonist RC-3095 on the growth of human pancreatic cancer cells in vivo and in vitro. *Cancer Res* **54**: 1035–1041

Robberecht P, De Neef P, Waelbroeck M, Camus J-C, Scemama J-L, Fourmy D, Pradayrol L, Vaysse N and Christophe J (1988) Secretin receptors in human pancreatic membranes. *Pancreas* **3**: 529–535

Sankaran H, Goldfine ID, Deveney CW, Wong K and Williams JA (1980) Binding of cholecystokinin to high affinity receptors on isolated rat pancreatic acini. *J Biol Chem* **255**: 1849–1853

Scemama J-L, Zahidi A, Fourmy D, Fagot-Revurat P, Vaysse N, Pradayrol L and Ribet A (1986) Interaction of [$^{125}$I]-Tyr$^4$-bombesin with specific receptors on normal human pancreatic membranes. *Regul Pept* **13**: 125–132

Schaffalitzky de Muckadell OB and Fahrenkrug J (1976) Preparation of $^{125}$I-labelled synthetic porcine secretin for radioimmunoassay. *Scand J Clin Lab Invest* **36**: 661–668

Schrenck TV, Moran TH, Heinz-Erian P, Gardner JD and Jensen RT (1988) Cholecystokinin receptors on gallbladder muscle and pancreatic acinar cells: a comparative study. *Am J Physiol* **255**: G512–521

Silvente-Poirot S, Dufresne M, Vaysse N and Fourmy D (1993) The peripheral cholecystokinin receptors. *Eur J Biochem* **215**: 513–529

Singh P, Townsend CM, Poston GJ and Reubi J-C (1991) Specific binding of cholecystokinin, estradiol and somatostatin to human pancreatic cancer xenografts. *J Steroid Biochem Mol Biol* **39**: 759–767

Smith JP, Solomon TE, Bagheri S and Kramer S (1990) Cholecystokinin stimulates growth of human pancreatic adenocarcinoma SW-1990. *Dig Dis Sci* **35**: 1377–1384

Smith JP, Kramer ST and Solomon TE (1991) CCK stimulates growth of six human pancreatic cancer cell lines in serum-free medium. *Regul Pept* **32**: 341–349

Smith JP, Liu G, Soundararajan V, McLaughlin PJ and Zagon IS (1994) Identification and characterization of CCK-B/gastrin receptors in human pancreatic cancer cell lines. *Am J Physiol* **266**: R277–R283

Tang C, Biemond I, Appel MJ, Visser CJT, Woutersen RA and Lamers CBHW (1995*a*) Gut peptide receptors in pancreata of azaserine-treated and normal control rats. *Carcinogenesis* **16**: 2951–2956

Tang C, Biemond I and Lamers CBHW (1995*b*) Localization and quantification of cholecystokinin receptors in rat brain with storage phosphor autoradiography. *Biotechniques* **18**: 886–889

Tang C, Biemond I, Appel MJ, Visser CJT, Woutersen RA and Lamers CBHW (1996) Expression of receptors for gut peptides in pancreata of BOP-treated and control hamsters. *Carcinogenesis* **17**: 2171–2175

Townsend CM Jr, Franklin RB, Watson LC, Glass EJ and Thompson JC (1981) Stimulation of pancreatic cancer growth by caerulein and secretin. *Surg Forum* **32**: 228–229

Upp JR, Singh P, Townsend CM and Thompson JC (1987) Predicting response to endocrine therapy in human pancreatic cancer with cholecystokinin receptors. *Gastroenterology* **92**: 1677

Virgolini I, Raderer M, Kurtaran A, Angelberger P, Banyai S, Yang Q, Li S, Banyai M, Pidlich J, Niederle B, Scheithauer W and Valent P (1994) Vasoactive intestinal peptide-receptor imaging for the localization of intestinal adenocarcinomas and endocrine tumours. *N Engl J Med* **331**: 1116–1121

© Cancer Research Campaign 1997

Wank SA, Pisegna JR and De Weerth A (1994) Cholecystokinin receptor family. Molecular cloning, structure, and functional expression in rat, guinea pig, and human. *Ann NY Acad Sci* **713**: 49–66

Williams JA, Bailey AC and Roach E (1988) Temperature dependence of high-affinity CCK receptor binding and CCK internalization in rat pancreatic acini. *Am J Physiol* **254**: G513–521

Yu D-H, Noguchi M, Zhou Z-C, Villanueva ML, Gardner JD and Jensen RT (1987) Characterization of gastrin receptors on guinea pig pancreatic acini. *Am J Physiol* **253**: G793–801

Yu D-H, Huang SC, Wank SA, Mantey S, Gardner JD and Jensen RT (1990) Pancreatic receptors for cholecystokinin: evidence for three receptor classes. *Am J Physiol* **258**: G86–95

© Cancer Research Campaign 1997